# The Integrated Care Workforce: What does it Need? Who does it Take?

**DOI:** 10.5334/ijic.7686

**Published:** 2023-07-07

**Authors:** K. V. Stein, N. Goodwin, E. Aldasoro, R. Miller

**Affiliations:** 1 Department of Public Health and Primary Care, Leiden University Medical Centre, The Netherlands; 2 International Journal of Integrated Care, The Netherlands; 3 Central Coast Research Institute, Faculty of Health and Medicine, University of Newcastle and Central Coast Local Health District, AU; 4 International Foundation for Integrated Care, The Netherlands; 5 Global Engagement for College of Social Sciences, University of Birmingham, UK

**Keywords:** integrated care workforce, competences, education and training, interdisciplinary

The health and care workforce was under pressure long before the pandemic due to the development of new technologies, recurring policy reforms and dwindling ressources. It has though taken the outcry in the aftermath of COVID-19 for researchers and policymakers to realise that something fundamental needs to change. In the integrated care literature and in this journal, the fact that integrated care implementation does not work without the involvement and support of the workforce has been a recurring theme, but also little heeded. As part of the Special Collection on the Building Blocks of Integrated Care, Stein (2016) already asked why building a competent workforce for integrated care is not yet a priority [1] and in reflecting on the impact of the COVID-19 pandemic on the workforce, we highlighted the need to take action and enable the workforce to become true partners in care through changes in education and training programmes, building trusted relationships and realising the potential of integrated care [2].

Concepts like the Quadruple Aim [3] or the WHO European Framework for Action for integrated health services delivery [4] have helped to shift the focus on workforce planning, education and training or role definitions for integrated care. But it doesn’t suffice to offer short-term, focussed trainings for specific programmes or initiatives. If we want the principles of integrated care to become engrained in the work ethic and culture of health and care, a lot more needs to happen. To name but a few examples, regulation and legal frameworks need to support sharing of information, data and accountability among teams and across professions and organisations; accreditation and professional institutions need to value and evaluate the competences for integrated care as an essential part of teaching and continuous development programmes; and funding and reimbursement schemes need to reflect the new ways of working and incentivise collaboration. The question of how technology can be designed and implemented to alleviate some of the pressures on the workforce and support integrated care instead of being seen as an added burden, is another crucial element for further exploration.

When discussing the future of the integrated care workforce, therefore, it becomes apparent that much needs to change. However, knowledge and experience of what this change looks like in practice, at which levels, and what the integrated care workforce and its support should be in the future are limited.

This special collection seeks to bring together papers that explore all facets of an integrated care workforce. It views the integrated care workforce as comprising more than just health and social care professionals and extending to carers, volunteers, community groups, as well as to people with lived experiences. As a Journal, we are open to being challenged on this viewpoint. Questions that are being explored in the articles of the special collection include:

- How do you teach integrated care, and the importance of workforce roles to support it? How do systems move towards a continuous learning organisation/system, which reinforces what you teach? Which theories, concepts, approaches, disciplines are necessary/relevant/useful to learn from?
- What support does the workforce need during the transition process from fragmented to integrated working? How do you empower existing professionals to work in team environments, engage in new relationships, and introduce them to new roles?
- What are some of the best examples which illustrate the development of a resilient, compassionate, sustainable and integrated workforce?

In their perspective paper for this special collection, Haring et al. highlight the many tensions that appear when opposing professional, organisations and system cultures are brought together to negotiate cooperation mechanisms and shared governance models. Rather than pretending that integrated care will automatically provide a common ground and foster understanding, they argue we should openly acknowledge conflicting values and interests and use these tensions to propel innovation, enabling the workforce to speak openly about their fears and pressures [5]. These tensions are often associated with new ways of working together in multi or interdisciplinary teams, applying more holistic approaches to care – like Positive Health – or implementing new tools like Project ECHO to support integration and coordination.

While there are some inspiring examples of successful workforce transformation in this special collection [e.g. 6], we also need to acknowledge that it took considerable time and effort to find and bring these together. Research and practice seem to treat the inclusion of the workforce similarly (if slightly more favourably) to the inclusion of people and communities, in that there is very little work reported on the subject matter [7]. Education and training programmes for the most part remain rigidly segregated both within and between health, including acute and primary care, social care, and other and emerging disciplines such as digital health. Digital health solutions are often developed and discussed in parallel to integrated care, rather than in combination with it, so that the workforce often feels technology adds an even bigger burden to their administrative tasks. Steele-Gray and colleagues (2021) have called for digital health to be seen and treated as a new member within an integrated care team, to ensure that the huge potential of digital solutions can actually be realised [8]. The same arguments have been made for other disciplines such as epidemiology and public health, health promotion, health justice, and many other separate – but intrinsically linked – work areas, with mixed results.

So, what will it take for the wokforce to be able to fulfil their roles and work collaboratively with people, families and communities, but also their colleagues across organisations and sectors? During the workforce based workshop at ICIC23 in Antwerp, the key messages were clear:

- First of all, we need to be clear who we actually mean when we talk about the integrated care workforce. There seems to be agreement that this includes health and social care professionals, and depending on the population, also other services like police or educators. In addition, change managers, administrators or accountants were mentioned, as they heavily influence the working environment of staff. The inclusion of volunteers, people with lived experience or family members within the workforce raised some concerns over putting too much pressure onto them, but also questions around ethics and regulations.
- To overcome the strong professionals and organisational cultures and identities, these should be acknowledged explicitly and used as the basis to build a common understanding, as they currently often work as a hidden barrier to change. Instead of seeing them as adversarial, people should be encouraged to build on what they already know and bring to the table.
- Education, training and continuous professional development programmes were seen as a key enabler to foster resilience, stress management and counteract burn out, but also build the new competences demanded by inter-disciplinary team work, digitalisation and task sharing. Specifically, behaviour change methods, self-determination theory or emergent planning were all mentioned as helpful tools to include in the training of professionals.
- The digital transformation was equally discussed as a possible time saver or time waster, as professionals are rarely involved in the design and implementation of applications. When calling for co-design in digital health, therefore, it is not only necessary to include people with lived experience, but also acknowledge professionals as end-users and thus important co-developers of digital solutions.
- As so often, there was a unanimous call for a stronger and better leadership and management approach during the entire transitional change process from status quo to integrated care. Integrated care is still being implemented as a task “on top of everything else” or “as a hobby”, and not given proper resources and importance.
- Storytelling, using positive and negative examples and narratives, as well as actively walking in somebody else’s shoes, e.g. through twinning or shadowing, were mentioned as powerful tools to support the workforce in understanding and endorsing the change.

Change towards a more integrated workforce has so far been elusive due to political, professional and academic inertia, but the global workforce shortage, especially in community, primary cand home care settings, is hopefully pressure enough to initiate the necessary changes in our education and training systems, regulations and professional accreditation schemes, as well as in the reimbursement and employment contracts. This special collection offers inspiration for professionals, managers, policymakers and academics alike, and is proof that change can happen against all odds and despite the tensions. Let’s turn this into a positive narrative, which nurtures and cares for the ones who nurture and care for us.
